# Update on the Molecular Genetics of Timothy Syndrome

**DOI:** 10.3389/fped.2021.668546

**Published:** 2021-05-17

**Authors:** Rosemary Bauer, Katherine W. Timothy, Andy Golden

**Affiliations:** ^1^Laboratory of Biochemistry and Genetics, National Institute of Diabetes, Digestive, and Kidney Diseases, National Institutes of Health, Bethesda, MD, United States; ^2^Timothy Syndrome Alliance, Gloucestershire, United Kingdom

**Keywords:** Timothy syndrome, arrhythmia, congenital heart defect, syndactyly, autism spectrum disorder, CACNA1C, Ca_v_1.2, variant

## Abstract

Timothy Syndrome (TS) (OMIM #601005) is a rare autosomal dominant syndrome caused by variants in *CACNA1C*, which encodes the α1C subunit of the voltage-gated calcium channel Ca_v_1.2. TS is classically caused by only a few different genetic changes and characterized by prolonged QT interval, syndactyly, and neurodevelopmental delay; however, the number of identified TS-causing variants is growing, and the resulting symptom profiles are incredibly complex and variable. Here, we aim to review the genetic and clinical findings of all published case reports of TS to date. We discuss multiple possible mechanisms for the variability seen in clinical features across these cases, including mosaicism, genetic background, isoform complexity of *CACNA1C* and differential expression of transcripts, and biophysical changes in mutant CACNA1C channels. Finally, we propose future research directions such as variant validation, *in vivo* modeling, and natural history characterization.

## Introduction

### Distinct Variants in the *CACNA1C* Gene Cause Timothy Syndrome

Timothy Syndrome (TS) (OMIM #601005) is an ultra-rare, autosomal dominant syndrome that was independently recognized in three case reports in 1992 and 1995 (1–3) and given its name in 2004 when a mutation in the calcium channel gene *CACNA1C* (12p13.33) was revealed as the molecular cause (4). The molecular defect was first identified in 13 children with syndactyly of the fingers and/or toes, prolonged QT interval as detected by an ECG, and neurological characteristics similar to autism-spectrum disorders. Because this was the eighth LQT syndrome gene to be identified molecularly, it was called LQT8 (4). As new disease-causing alleles were discovered though, the syndrome became much more complex, variably including congenital heart defects (CHDs), hypertrophic cardiomyopathy (HCM), facial dysmorphisms (including small teeth, low set ears, and flat nasal bridge), intermittent hypoglycemia and infections, and epilepsy. This review will explore the full spectrum of symptoms that have been identified in the years since the syndrome's original identification.

**Figure 1:**
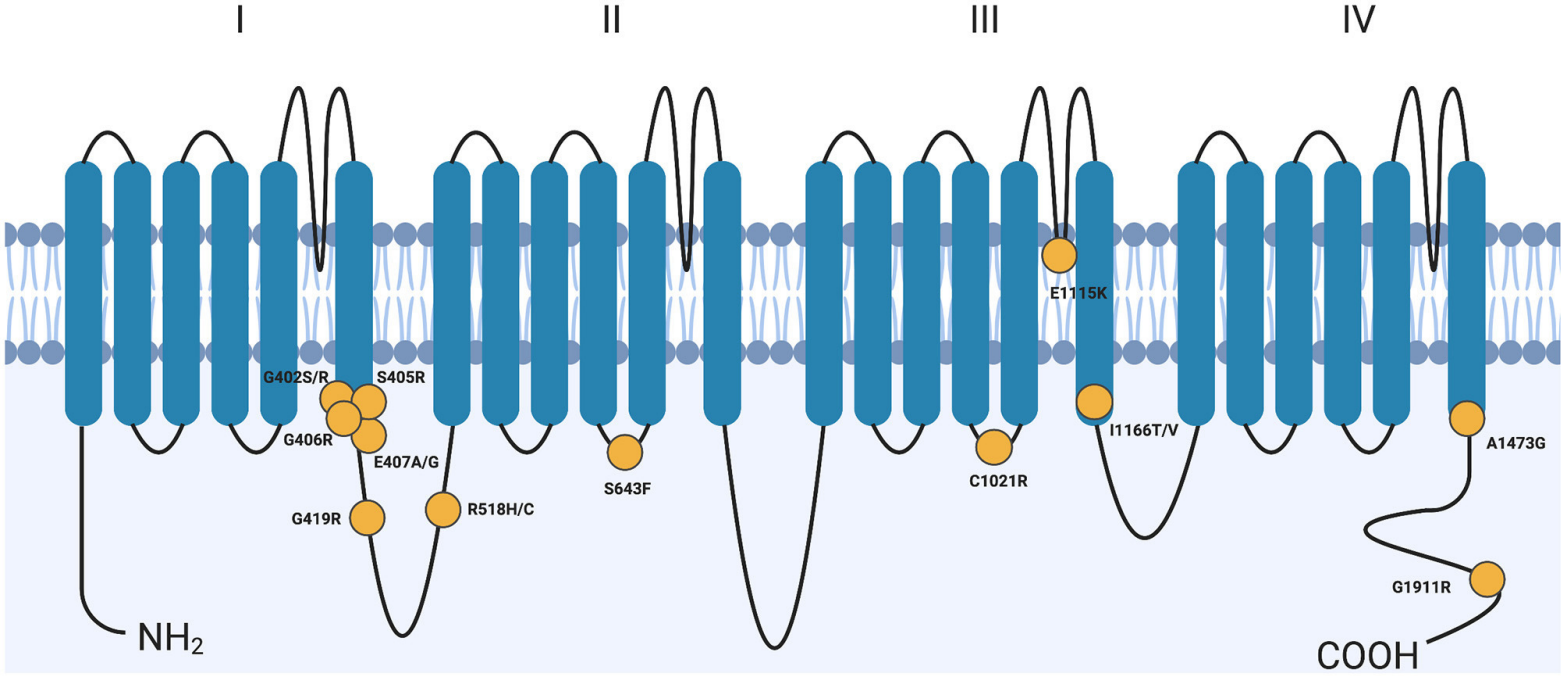
Variants across CACNA1C cause Timothy syndrome. CACNA1C is a 24-transmembrane-pass channel that consists of four repeats (I–IV) of 6 transmembrane segments (SI-S6). Yellow circles denote approximate locations of TS-causing variants. Though G406R is only shown once, it does represent two distinct mutations, one in mutually exclusive exon 8 or exon 8A. G, Glycine; R, Arginine; S, Serine; E, Glutamate; A, Alanine; C, Cysteine; H, Histidine; F, Phenylalanine; K, Lysine; I, Isoleucine; T, Threonine; V, Valine. Created with BioRender.com.

Calcium flux is essential for proper function of all cells. Defects in ion channel function, or channelopathies, are responsible for a number of diseases including many that result in cardiac arrhythmias and much more complex syndromes. Mutations in the *CACNA1C* gene have been linked to multiple arrhythmic syndromes, including Long QT Syndrome Type 8 (LQT8), short QT (SQT), and Brugada Syndrome [OMIM #618447 and 611875 (4–6)]. *CACNA1C* encodes the α1C subunit of the Ca_v_1.2 voltage-gated calcium channel (VGCC) that is expressed in many tissues of the body (4). The channel also associates with an intracellular β subunit and an extracellular α_2_δ subunit to form the fully functional channel complex; this review will focus on the α1C subunit since it is the primary pore-forming unit of the channel. The α1C subunit has 4 domains (I–IV), each with six transmembrane (TM)-spanning segments (S1–S6), that make up the large 24-transmembrane-pass channel (Figure 1).

## Timothy Syndrome Type 1

Splawski et al. (4) originally characterized 13 individuals when they reported the molecular cause of Timothy Syndrome. All 13 children had a missense variant in exon 8A of the *CACNA1C* gene resulting in a Gly406Arg amino acid change. This variant disrupts the 6th transmembrane helix (S6) in domain I of the α1C subunit (Figure 1) and results in a gain-of-function phenotype. This specific syndrome became known as TS Type 1 (TS1) when a second allele was discovered shortly after this initial report. TS was originally described and studied because of its association with a prolonged QT interval and had a relatively simple symptom profile of prolonged QT interval (QTc), syndactyly, autism, tooth decay, and facial dysmorphia.

## Timothy Syndrome Type 2

**Figure 2:**
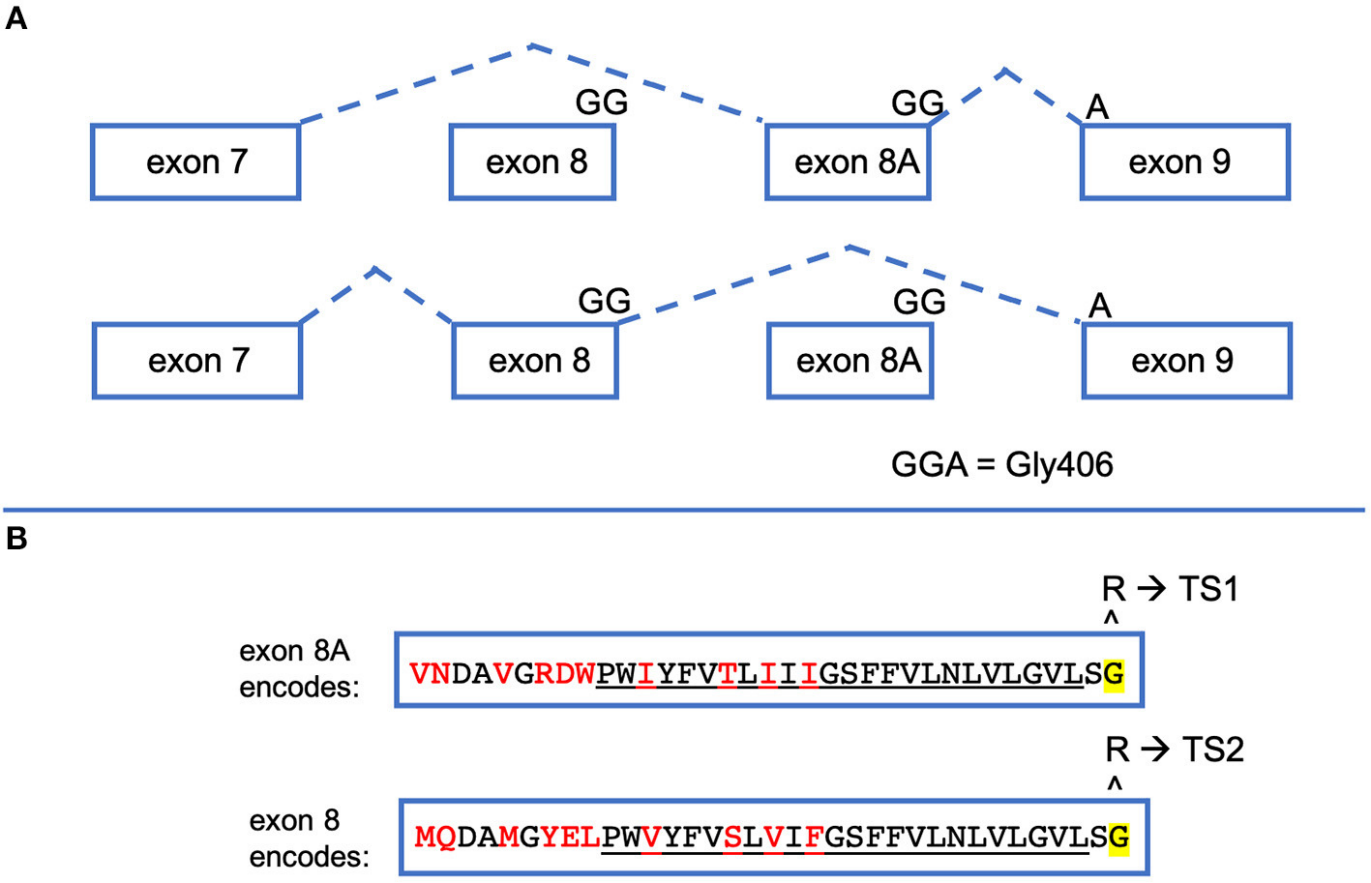
*CACNA1C* exon 8 and exon 8A are alternatively spliced. **(A)** When *CACNA1C* mRNA is processed, exon 7 is spliced to either of two mutually exclusive exons, exon 8 or exon 8A, which is then spliced to exon 9. Exons 8 and 8A are the exact same size (104 bp). The final two nucleotides of exon8/8A and the first nucleotide of exon 9 encode Gly406, the amino acid most commonly implicated in TS. **(B)** Exons 8 and 8A produce slightly different protein products. Both encode 34 amino acids, differing by the 10 shown in red. The underlined amino acids make up the S6 segment of the first CACNA1C repeat. If the Gly406Arg variant is caused by nucleotide changes in exon 8A, the resulting phenotype is TS1. If the nucleotides in exon 8 are altered to change Gly406 to Arg, TS2 results.

The *CACNA1C* gene has numerous splice forms, but important to TS is the alternative splicing of exon 8 and exon 8A. Some confusion in the field exists because different groups published the gene structure of *CACNA1C* but denoted exon 8 and an alternatively spliced exon 8A in different orientations to each other (GenBank Z26263) (4, 7, 8). The paper by Splawski et al. has been accepted by most, if not all, publications that refer to *CACNA1C*, putting exon 8 upstream of exon 8A (Figure 2). Exon 8 and 8A are both 104 nucleotides in length, coding for 34 amino acids. They differ in 10 of those amino acids, but both exons code for a Gly at residue 406. mRNAs bearing these alternatively spliced exons are differentially expressed. In organs most notably affected by TS such as brain and heart, exon 8-bearing mRNAs are about 4 times more abundant than exon 8A-bearing mRNAs (9). In 2005, a single child was reported with a Gly406Arg variant in exon 8 (9). The disease caused by Gly406Arg in exon 8 became known as TS Type 2 (TS2). Interestingly, children with an exon 8A variant resulting in Gly406Arg are less-severely affected than children with the same missense variant in exon 8, which likely is due to the difference in expression levels in the heart and brain. TS2 individuals do not present with syndactyly but do variably experience other extra-cardiac symptoms, the only unique symptom of TS2 not seen in TS1 being hip dysplasia. Since the original 2005 publication, more children with Gly406Arg in exon 8 have been identified and these TS2 children also do not present with syndactyly but do present with many symptoms originally associated with TS. These individuals also appear to have worse clinical outcomes than those with the 8A variant, often dying at a younger age. It has come to be accepted that all Gly406Arg variants cause TS regardless of which exon bears this missense variant. Contrary to an opinion expressed in a recent report (10), it still seems clinically useful to note the molecular distinction between exon 8 and 8A, as it may still have some predictive value for the natural history of this disease (i.e., less severe disease and hip dysplasia in individuals with exon 8 Gly406Arg).

It should be noted that LQT8 also stands alone as a cardiac arrhythmia syndrome. These individuals do have variants in the *CACNA1C* gene distinct from those described throughout this review. A significant number of individuals with a *CACNA1C* variant do present with non-syndromic LQT8, but do not have Timothy Syndrome (11–13). To our knowledge, there is no quantification of non-syndromic LQT8 individuals to compare with those affected by TS.

## Atypical Timothy Syndrome

This review serves to highlight the other missense variants that have been identified as TS-causing alleles as well as to stress that sequencing only exons 8A and 8 are no longer the best approach when a TS diagnosis is suspected. These alleles are often referred to as Atypical TS (ATS) alleles (14). This review proposes new nomenclature for how to refer to these distinct new variants as they arise.

In addition to the Gly406Arg variant from exon 8, another missense variant was identified in exon 8 in a single child, resulting in a Gly402Ser change and was initially referred to as TS2 (9). This child did not have syndactyly but did have some neurological deficits similar to TS. It should be noted however that these neurological deficits developed after the child experienced a sudden cardiac arrest. More recently, two siblings with the same Gly402Ser variant were reported to be LQT-only TS2 (15). Their father was mosaic for the variant. Interestingly, they had partial syndactyly. In another report, a Gly402Ser individual was diagnosed as only having the cardiac arrhythmia LQT8 and not a syndromic disorder; alternatively, this individual could be mosaic for the variant (16). So far, this variant has only been observed in exon 8. Since there are only four published cases of Gly402Ser variants in the literature, it remains to be resolved whether this variant is causative of classic TS or a non-syndromic LQT. The Gly402Ser variant has recently been placed into the larger group of ATS alleles. ATS variants essentially include all missense variants that do not result in the single amino acid change Gly406Arg (TS1 and TS2). We agree that Gly402Ser (exon 8) should be classified as an ATS allele since it is quite symptomatically distinct from TS2. Currently, OMIM only lists these two variants for Timothy Syndrome—Gly406Arg and Gly402Ser.

Since 2005, at least 12 other missense variants have been identified as TS-causing alleles (Table 1). These were all published as case reports and as such, the number of individuals with each reported variant is usually one. One theme that seems to arise in ATS cases is variability in disease presentation. This variability could be due to mosaicism, genetic background, or transcriptional regulation leading to variable expression levels, all of which are discussed later in this review. Below is a brief summary of each of these novel variants since the publication of the original TS1 and TS2 papers from 2004 and 2005. Since they each vary in their presentation, it is difficult to assemble them into distinct groups.

**Table 1:** CACNA1C variants causative of Timothy syndrome.

**CACNA1C TS variant**	**Number of children**	**LQT**	**Syndactyly**	**NDD**	**Hypoglycemia**	**Other phenotypes**	**Reference(s)**
Gly402Ser (exon 8)	1	Yes	No				(9)
Gly402Ser (exon 8)	2	Yes	Partial				(15)
Gly402Ser (exon 8)	1	Yes	No	No			(16)
Gly402Arg (exon ?)	1	Yes					(17)
Ser405Arg (exon ?)							(17)
Gly406Arg (exon 8A)	13	Yes	Yes				(4)
Gly406Arg (exon 8A)	1	Yes					(18)
Gly406Arg (exon 8A)	1	Yes	No	No		Mosaic?	(19)
Gly406Arg (exon 8A)	1	Yes	Yes			Maternal mosaicism	(20)
Gly406Arg (exon 8A)	1	Yes	Yes			Paternal mosaicism	(21)
Gly406Arg (exon 8A)	1	Yes	Yes				(22)
Gly406Arg (exon ?)	1	Yes	Yes	No	No	Mosaic	(23)
Gly406Arg (exon 8)	1	Yes	No			Hip dysplasia	(9)
Gly406Arg (exon 8)	1	Yes					(10)
Glu407Gly	1	Yes	Yes	Yes		Epilepsy	(24)
Glu407Ala; PKP2	1						(25)
Gly419Ser	1	Yes	Yes	Yes			(26)
Arg518Cys	5 familial, 1 additional outside of family	Yes	No	No	No	HCM	(27)
Arg518His	1	Yes				HCM	(27)
Ser643Phe	1	Yes	No	Yes		Seizures	(28)
Cys1021Arg							(17)
Arg1024Gly	1	No	Yes				(29)
Glu1115Lys	1	Yes	No	Yes	Hyper	Seizures	(30)
Ile1166Thr	1	Yes	Clinodactyly				(31, 32)
Ile1166Thr	1	Yes	No				(11)
Ala1473Gly	1	Yes	Yes		Yes	Hip dysplasia	(14)
Gly1911Arg	1	Yes	No	Yes		Many	(33)
Before sequence analysis	1	Yes	Yes				(2)
Before sequence analysis	1	Yes	Yes				(1)
Before sequence analysis	3	Yes	Yes				(3)

*LQT, long QT; NDD, neurodevelopmental delay.*

*All of the variants in this Table are based on the cDNA sequence used by Splawski et al. [(4); GenBank Accession number Z34815] except for the Arg1024Gly variant, which was based off of a longer isoform of CACNA1C [(29); GenBank Accession number NM_199460]*.

In 2012, Gillis et al. reported a single individual with an Ala1473Gly missense variant (14). Like Gly406, this variant resides on the intracellular portion of S6, domain IV (Figure 1). At birth, this child had dysmorphic facial features, sparse hair growth, bilateral hip dislocation, joint contractures in the arms, and syndactyly of fingers and toes on both hands and both feet. The child also failed many neurological and reflex tests at birth. This individual would go on to have seizures, episodes of hypoglycemia and hypocalcemia, apnea, bilateral hip dysplasia, cortical blindness, and severe neurodevelopmental delay (14).

An Ile1166Thr missense variant was identified in two children by three different groups, and like Gly406 and Ala1473, is also on the intracellular portion of S6 but in domain III (11, 31, 32). One child presented with 2:1 AV block shortly after birth with QT-interval prolongation, patent ductus arteriosis (PDA) and left atrial enlargement (32). An ICD was implanted. He was later found to have numerous structural cardiac defects and at 5 months of age began a history of seizures. Consistent with TS, this child had facial dysmorphisms, joint hypermobility, clinodactyly, hypotonia, and severe dental abnormalities leading to the extraction of 20 teeth before the age of 3 (31, 32). This child died at 3 years 8 months after admission for respiratory failure, hypotension, and dehydration. The second child identified suffered sudden cardiac death at age 1 prior to molecular testing, but presented with severely prolonged QT interval, AV block, ductus arteriosis, HCM, and syndactyly. The *CACNA1C* mutation was identified post-mortem, and a diagnosis of TS was thus presumed (11). Interestingly, the position of the missense variants, Gly406Arg, Gly402Ser, Ala1473Gly, and Ile1166Thr, all occur near the cytoplasmic face of the S6 segment of their respective domains in the CACNA1C structure (Figure 1).

An individual was identified with the missense variant Gly1911Arg in the C-terminal cytoplasmic tail of the CACNA1C channel [Figure 1 (33)]. Unlike the individuals with variants in the S6 segments, this individual developed normally until 30 months of age, when he began experiencing seizures. The seizures were typically accompanied by a loss of consciousness, and at age 5 a seizure was accompanied by monomorphic ventricular tachycardia which required resuscitation. The patient was then diagnosed with prolonged QTc. Follow-up exams at ages 12 and 17 revealed subtle muscular degeneration, microcephaly, dental abnormalities, and facial dysmorphisms including those common in TS such as flat nasal bridge (33).

The variant Arg518Cys was identified as the disease-causing mutation in a family with multiple affected individuals suffering from QTc prolongation, HCM, SCD, and other congenital heart defects (CHDs) (27). None of these family members had syndactyly, cognitive impairments, facial dysmorphisms, or any extracardiac phenotypes common to a TS diagnosis, so the authors designated this disease presentation “cardiac-only TS” (COTS). Armed with a new, previously-unreported TS-causing variant, they screened samples from a cohort of patients with idiopathic LQTs for this change. Through this screen they identified another individual with the Arg518Cys variant and one with Arg518His in CACNA1C. These individuals had family histories of QTc prolongation and HCM and both variants cosegregated with the cardiac phenotypes (27). The nomenclature assigned to these cases, COTS, is somewhat confusing especially considering there are variants within the *CACNA1C* gene that cause non-syndromic LQT8, which is also a cardiac-only disease. COTS is distinct from non-syndromic LQT8 in that there are structural heart defects in addition to the prolonged QTc. We propose that cases previously designated COTS be considered ATS to further differentiate them from non-syndromic LQT8. This will also be discussed in terms of mosaicism later in this review.

A 2018 study following the treatment outcomes of many TS individuals identified three previously unreported missense mutations: Gly402Arg, Ser405Arg, and Cys1021Arg (17). The Gly402 and Ser405 variants, like many discussed earlier, reside on the intracellular side of transmembrane segment S6. Gly402Arg was present in two unrelated patients, but symptoms were only described for one. The affected individual presented with severely prolonged QTc and neurodevelopmental delay. Two related individuals were diagnosed with the missense allele Ser405Arg. Both of these cases included prolonged QTc and syndactyly, but only one included neurodevelopmental delay. The individual with the missense variant Cys1021Arg exhibited the most complex symptom profile, presenting with long QTc, syndactyly, neurodevelopmental delay, facial abnormalities, baldness, and intermittent hypoglycemia (17).

The TS symptom profile was further complicated when Kosaki et al. reported an individual with the missense mutation Arg1024Gly who presented with many common TS phenotypes but *not* QTc prolongation (29). In addition to bilateral cutaneous syndactyly, this individual experienced delayed growth and neurodevelopment, upper limb joint contractures, several seizure-like episodes, thin scalp hair, and facial dysmorphisms (29), all consistent with other cases of TS.

A 14-year-old boy with a prolonged QTc, facial dysmorphisms, neurodevelopmental delay, a history of seizures, but without syndactyly, was diagnosed with TS when his causative mutation, Ser643Phe in CACNA1C, was identified (28). This variant is positioned on the cytoplasmic loop between S4 and S5 of domain II in the CACNA1C channel. Variants associated with LQT8 have also been identified on cytoplasmic loops in domain II, in the loop between domain II and III, and on the C-terminal cytoplasmic tail of this channel (11, 36, 37). This is the first case report of a similarly-located variant that resulted in a TS diagnosis. Although this individual's symptom profile matches that of TS, the relatively late age of diagnosis (14 years old) is notable.

A 14-year-old was identified to have the variant Glu1115Lys after undergoing genetic testing for idiopathic long QT (30). Although the case report does not suggest a TS diagnosis, the patient presented with prolonged QTc, autism spectrum disorder, seizures, and hyperglycemia (rather than hypoglycemia). The Glu1115Lys variant has also been observed in an individual with Brugada Syndrome (38). How some individuals present with such different symptoms and at such a later age than most TS individuals remains unknown, but some possibilities are discussed later.

Two recent case reports suggest that mutations in residues located near Gly406 (that causes TS1) can also cause TS. A Glu407Ala missense variant (25) and a Glu407Gly missense variant (24) were identified in two different children. This last case was most like TS1 with reported syndactyly and LQT, but also included epilepsy. The individual with Glu407Ala presented with mild learning disabilities, epilepsy, and bilateral cutaneous syndactyly, but importantly also has a missense variant in the gene *PKP2* (25). PKP2 is a component of desmosomes and has been shown to modulate L-type calcium channels (39). The patient was 22 years old at the time of diagnosis, so the secondary genetic change may somehow modulate disease presentation.

Most recently, a Gly419Arg variant was reported The affected individual presented with bilateral hand and foot syndactyly, PDA, left ventricular non-compaction, ventricular preexcitation, mild neurodevelopmental delay, and prolonged QT interval. Interestingly, after surgical closure of the PDA at age 3 months and correction of hand syndactyly at age 2, this individual experienced no symptoms until he suffered syncope and pre-excited atrial fibrillation at age 15. An ICD was implanted at age 17. The individual is now 23 years old. It is important to note that he has two additional *de novo* variants: Glu131Gln in *TOP1*, a DNA topoisomerase, and Gly2493Arg in *ITPR1*, a channel that releases calcium from the ER upon inositol 1,4,5-triphosphate binding (26). Although the consequences of these variants are unknown, they could provide important context for understanding this case.

## How Does a Variant Arise?

Autosomal dominant genetic diseases, like LQT1 for example, are passed on from generation to generation because the presentation is most often sub-lethal and can be managed with medication. Additionally, some may not even recognize that they had the disorder. Thus, the mode of inheritance for such disorders is genetic—one parent passes the mutation onto their progeny with an inheritance likelihood of 50% for each child. Due to the much higher severity of disease, TS does not get passed on by an affected parent in this way because affected individuals rarely survive to reproductive age.

*De novo* genetic changes are defined as genetic alterations that are present for the first time in one family member as a result of a variant in a germ cell (egg or sperm) of one of the parents, or variants that arise in the fertilized egg itself during early embryogenesis (https://www.cancer.gov/publications/dictionaries/genetics-dictionary/def/de-novo-mutation). *de novo* variants arise due to errors in DNA replication and account for most of the normal genetic variation that we observe in all organisms. Since TS is such a harmful disease, it is likely that most TS children acquire the variant *de novo* rather than inherit it from an affected parent. So, *de novo* mutations may arise during gametogenesis of a parent such that the mutation is present in the mother's egg or father's sperm, and the resulting embryo bears the mutation upon fertilization. *de novo* mutations acquired in parental germ cells are present in all of the resulting embryo's cells and tissues, leading to a fully heterozygous individual for the variant. The parent can be considered mosaic (having genetically distinct cells within a single individual) for the variant, since only one cell type (gamete) is affected, but the child may not be. It is becoming clear that *de novo* mutations can also appear post-zygotically, leading to mosaicism of the developing embryo (40). Thus, the affected child is mosaic and expresses the variant in some of their cells and organs. If this error were to happen early, more cell lineages would have the error; if it occurred later in development, fewer cells and organs may contain that mutation. Here we discuss these modes of inheritance and how they could be essential in understanding variability in TS disease presentation.

### Inheritance From Mosaic Parents

Parental germline mosaicism appeared to be the case for a Gly406Arg child whose father carried the same variant in 16% of his sperm (41). This low level of mosaicism was possible to detect through cloning and sequencing multiple PCR-amplified products from the father's sperm. The father had QTc prolongation but no other TS-like symptoms. Without being able to biopsy various tissues from a living mosaic individual, we can only presume that this father did not have the TS variant expressed in many other organs other than his heart and gametes.

Since a parent provides a haploid gamete for the creation of a zygote, a mosaic parent either passes on the wildtype copy of the gene or the variant version. In this scenario, the parent is mosaic, but the child is fully heterozygous for the variant from the time of fertilization; that child will maintain the variant in every tissue and organ. We speculate that the TS children born of a mosaic parent likely present with more severe disease than a *de novo* child who acquires the variant during embryogenesis. In a child who acquires the variant during embryogenesis, fewer organs and tissues may bear this variant. For these reasons, it would be important to determine whether a parent is mosaic and to consult a genetic counselor if the parents are considering additional children in the future. If some of either parents' gametes carry the TS variant, that parent could pass the TS allele onto future progeny.

### Embryonic *de novo* Mutation

Post-zygotic point mutations are an under-recognized source of *de novo* genomic variation (40). Some *de novo* children may also be mosaic and that may explain, in part, why not all TS children report deficits in all tissues. Recently, a child who was initially negative for any known variants in *CACNA1C* had their lymphocytes re-analyzed by Next Generation Sequencing (NGS). A Gly406Arg variant was identified in 18% of the sequencing reads. This child had an “incomplete phenotype” in that only LQT and syndactyly were noted, and none of the other common TS phenotypes (23). The authors proposed that there may be additional cases of incomplete TS that go unnoticed by standard gene panel Sanger sequencing. NGS has the benefit of >100-fold coverage of the genome such that mosaicism can be readily detected, even if only present in a few sequencing reads.

## Is There an Explanation for the Variability in Presentation of TS?

There is an incredible amount of phenotypic variability among individuals bearing variants in the *CACNA1C* gene and TS no longer exclusively applies to children with both cardiac arrhythmias and syndactyly. What remains to be resolved is whether non-syndromic LQT8 is a distinct disease or just another mosaic presentation of TS. The existence of a variant in the *CACNA1C* gene is not enough to assign a diagnosis of TS. But why is it that all individuals with variants in *CACNA1C* do not present with identical phenotypes? There are many possibilities for this observation, as is true for many monogenic diseases.

**Figure 3:**
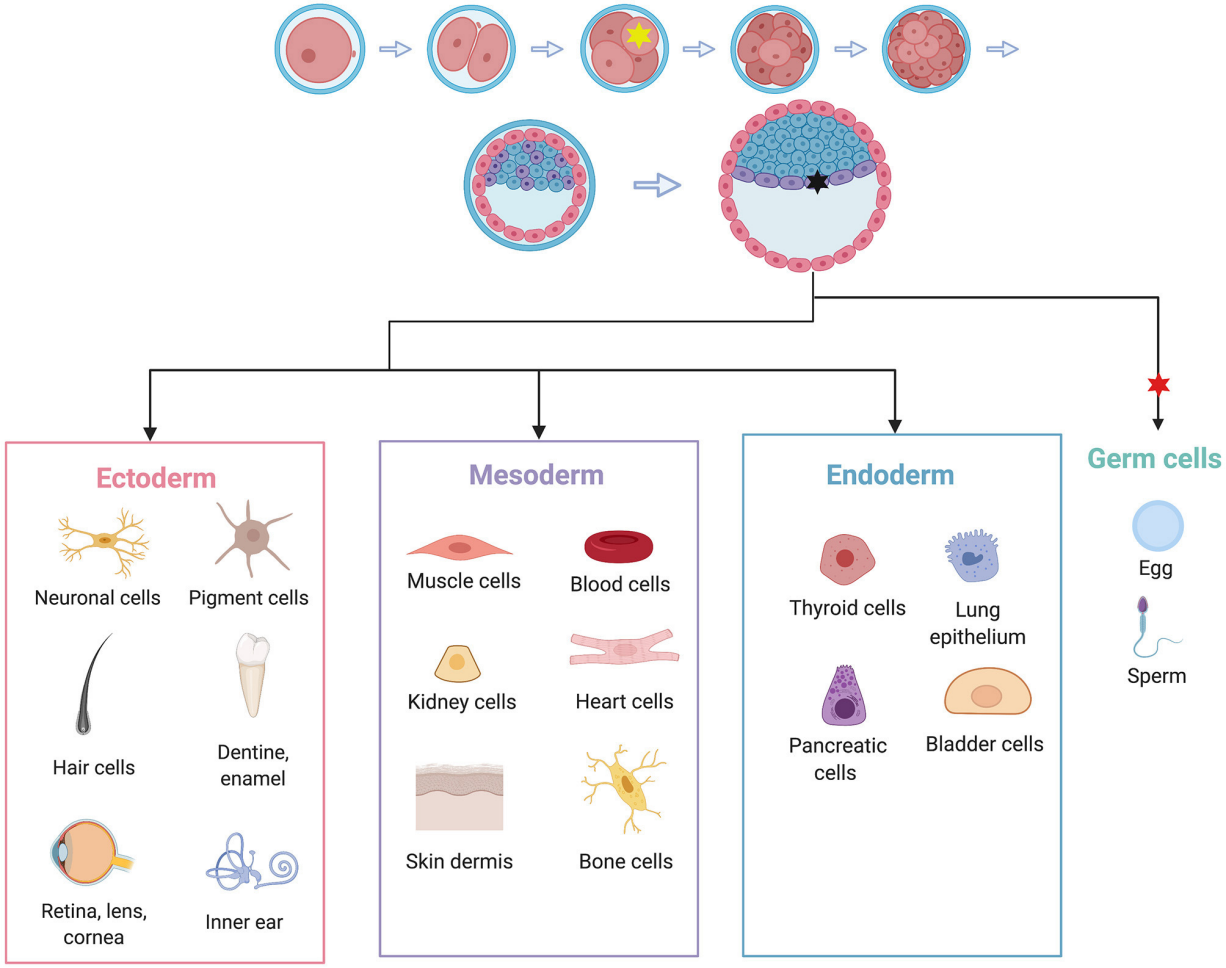
Embryonic *de novo* mutations can result in highly variable phenotypes due to mosaicism. Individuals who acquire a mutation at the 4-cell stage, represented by the yellow star, may experience symptoms in all cell types (given that the variant in question has equal ability to affect all cell types). A variant acquired at the gastrula stage, represented by the black star, may only affect one germ layer and therefore a more restricted subset of cell types. In this example, the black mutation may only affect heart, muscle, kidney, skin, and bones, but not neuronal tissue. In the case of TS, this is a possible mechanism for cases that have symptoms restricted to LTQ and syndactyly. A variant that arises after germ cells have been set aside, represented by the red star, may only affect germ cells, and therefore may not cause phenotypes in the individual, but may affect the individual's children. Adapted from “Human Embryonic Stem Cell Differentiation,” by BioRender.com (2020). Retrieved from: https://app.Biorender.com/biorender-templates.

One explanation is through mosaicism as discussed in the previous section. Individuals who inherit a variant at fertilization will likely have a more severe disease presentation than those who acquire it at some point during embryonic development. Additionally, depending on the timing and location of an embryonic *de novo* variant, certain germ layers or cell lineages could be differentially affected (Figure 3). This could explain how different individuals with TS present with symptoms restricted to different sets of tissues.

A second likely explanation for variability in presentation is that each TS child has a very different genetic background, which can contribute to expressivity of the symptoms. For example, the young woman diagnosed with the *CACNA1C* variant Glu407Ala at age 22 had a secondary variant in PKP2 (25). Age 22 is much older than most TS individuals are diagnosed, and much older than many survive; it is likely that her presentation of TS is influenced by the presence of a second mutation. Both *CACNA1C* and *PKP2* were included on the rhymologic gene panel run in this individual's case, making it a simple case of two pathogenic mutations. If all individuals with TS underwent whole-exome or whole-genome sequencing in relation to their parents and/or siblings, multiple genetic changes might be revealed that contribute to complex genetic profiles influencing each child's presentation of disease. This possibility exists for most, if not all, inherited diseases.

A third explanation could involve the many different isoforms of the CACNA1C protein. Besides the use of alternative exons 8 and 8A, there is evidence that hundreds of alpha-subunit isoforms exist due to alternative splicing (42). The use of so many different isoforms of CACNA1C make it difficult to establish simple patterns of disease. It may be that ATS variants do not phenocopy the Gly406Arg variant because certain isoforms are expressed more or less abundantly in various tissues, and some threshold of mutant CACNA1C channels is tolerable. Studies of the Gly406Arg allele in adult guinea pig ventricular myocytes (aGPVMs) showed that cells could tolerate a certain proportion of mutant CACNA1C channels. A low proportion of mutant channels caused action potentials (APs) to be only slightly prolonged; once this tolerance threshold was crossed, APs became unstable and pushed cells into an “arrhythmogenic” state. In aGPVMs, this threshold was about 12% for Gly406Arg and about 40% for Gly402Ser (34). This result could be essential in understanding the differences in pathogenesis of TS1 and TS2, and more precise knowledge of transcript expression by tissue could inform the discussion of other, non-exon 8 or 8A alleles.

Alternatively, it may not be the tissue-specific expression levels that matter but that a given tissue, organ, or cell type is more sensitive to an alteration in function of the CACNA1C channel. Each of the missense variants that have been associated with TS compromise the function of the channel, and perhaps to different extents and for different biophysical reasons. It is possible that some cell types are more tolerant to this alteration in function than others, and it is the cell types or organs that are most sensitive that result in disease symptoms. This may explain why numerous variants throughout the *CACNA1C* gene result in non-syndromic LQT8 and manifest as a disease that has only one symptom. Perhaps the heart is the most sensitive tissue to any alteration in function of the CACNA1C channel and that is why so many seemingly unrelated missense variants throughout the gene are associated with LQT8.

One could hypothesize that the reason so few TS missense variants have been identified to date is that these variants disrupt channel function more dramatically than the non-syndromic LQT8-causing variants. Other organs besides the heart may not be able to tolerate this alteration in function, and this is why we observe syndactyly, neurodevelopmental delays, hip dysplasia, facial abnormalities, hypoglycemia, and other symptoms. We would go so far as to predict that there may be *CACNA1C* variants that result in any one of these phenotypes without appearing multi-system in nature. For example, there could be hypoglycemic children with *CACNA1C* variants that have no other TS-like phenotypes. The Arg1024Gly variant (29) supports such a prediction in that this individual has few TS phenotypes, such as syndactyly, but lacks QTc prolongation. This could be a case of mosaicism or a case in which the amino acid change itself is not as detrimental to heart tissue as it is elsewhere. Additionally, cases previously designated as COTS (Arg518Cys, Arg518His) (27) could be interpreted as ATS variants that have effects restricted to the heart, either through embryonic *de novo* mosaicism or due to higher heart-specific susceptibility to changes to this specific residue.

There may also be *CACNA1C* variants that are so poorly tolerated that these children die at a very young age and are never diagnosed with TS. That certainly may be true for some exon 8 Gly406Arg children. Genetic variants that result in very early death may 1 day be diagnosed as more clinicians perform Whole Exome Sequencing (WES) on infants that die of SIDS (43).

It can be argued that Katherine Timothy and the cardiologists she worked alongside selected a specific subset of TS children in their landmark 2004 publication in which they first molecularly identified the genetic lesion responsible for TS (4). Because they only sequenced the *CACNA1C* gene from children presenting with both LQT and syndactyly, it is now clear that they selected for a homogenous group of children. Sixteen years later and after many molecular genetic case reports of ATS children, it is apparent that children presenting with *CACNA1C* variants and a subset of the “classical” TS phenotypes should also receive the diagnosis of TS. As with many syndromes, not every individual bearing a specific variant presents with all of the same phenotypes, yet most clinicians readily accept that their diagnosis is correct. The acceptance that many distinct variants in *CACNA1C* can cause TS may prompt clinicians in this field to rethink how they categorize these seemingly different types of TS.

## What are the Mechanisms of Disease?

In addition to the previously-discussed sources of variability, biophysical properties of the variant channels themselves could also account for differences in disease presentation. To date, all individuals with TS are heterozygous for their variant. TS-causing mutations are gain-of-function alleles of *CACNA1C*, prolonging the cardiac action potential (AP) and therefore the QT interval (QTc). Various groups have investigated the electrophysiological effects of different mutations on CACNA1C function and have found a few distinct mechanisms of dysfunction. The classical mechanism of TS pathogenesis is a loss of voltage-dependent inactivation (VDI) of the channel, thereby causing an increase in the maximum flow of Ca^2+^ through the channel (peak current density) in a given opening event (4, 9, 33). Alternative mechanisms are characterized by a decrease in peak current density and commonly a decrease in degree of depolarization necessary to activate the channel (11, 26–28, 30, 32). Here we review the electrophysiological studies of CACNA1C channel variants in model systems to date (Table 2).

**Table 2:** Electrophysiological effects of CACNA1C variants in model systems.

**CACNA1C TS variant**	**Study system**	**Electrophysiological findings**	**Reference(s)**
Gly402Ser exon 8	*Xenopus* oocytes	Impaired VDI	(9)
Gly402Ser exon 8	HEK293 cells	Impaired VDI, decreased F_CDI_	(34, 35)
Gly406Arg exon 8A	CHO cells, *Xenopus* oocytes	Impaired VDI	(4)
Gly406Arg exon 8A	HEK293 cells	Impaired VDI, decreased CDI_max_	(34, 35)
Gly406Arg exon 8	*Xenopus* oocytes	Impaired VDI	(9)
Gly406Arg exon 8	HEK293 cells	Impaired VDI, decreased CDI_max_	(34, 35)
Gly419Arg	HEK293 cells	Decreased V_1/2_ of activation, increased window current	(26)
Arg518Cys	HEK293 cells	Reduced peak current density, reduced channel localization to membrane, decreased V_1/2_ of inactivation, increased window current, impaired VDI	(27)
Arg518His	HEK293 cells	Reduced peak current density, decreased V_1/2_ of activation and inactivation, increased window current, impaired VDI	(27)
Ser643Phe	HEK293 cells	Reduced peak current density, decreased V_1/2_ of activation, impaired VDI	(28)
Glu1115Lys	TSA201 cells	Converted channel to non-selective monovalent cation channel with inward Na^+^ current and outward K^+^ current	(30)
Ile1166Thr	HEK293 cells	Reduced peak current density, decreased V_1/2_ of activation, decreased SSI	(11, 32)
Ile1166Thr	*Xenopus* oocytes	Decreased V_1/2_ of activation, decreased SSI	(11)
Gly1911Arg	HEK293 cells	Impaired VDI, decreased SSI, increased window current	(33)

### Variants Resulting in the Classical Mechanism: Gly402Ser, Gly406Arg, Gly1911Arg

The first descriptions of TS included electrophysiological studies done in Chinese hamster ovary (CHO) cells and *Xenopus* oocytes (4, 9). Studies in both systems revealed that expression of a transgene containing the Gly406Arg variant in exon 8A results in CACNA1C channels that have impaired VDI, leading to prolonged opening of the channel and subsequently increased Ca^2+^ flux through the channel (4). The same group later expressed constructs containing the Gly406Arg mutation in exon 8 as well as Gly402Ser in exon 8 in *Xenopus* oocytes. Both of these mimicked the original exon 8A mutation: loss of VDI which produced sustained inward Ca^2+^ currents (9).

In addition to these early studies, many groups have since investigated the original TS mutations in great detail and in more systems. Expression of the Gly406Arg variant in HEK293 cells led to a loss of VDI (35). Although they reported that Gly406Arg has no effect on calcium-dependent inactivation (CDI) of CACNA1C, Dick et al. later carefully examined this parameter. They expressed transcripts encoding Gly406Arg-exon 8A, Gly406Arg-exon 8, or Gly402Ser-exon 8 of CACNA1C in HEK293 cells and found that all three of these caused a large decrease in CDI when compared to WT (34). They were also able to elucidate two contrasting methods of CDI loss in the two different missense changes: Gly406Arg in either exon led to a decrease in CDI_max_, a measure that is a function of channel gating and a result which reflects an increase in channel opening, whereas Gly402Ser led to a decrease in F_CDI_, a measure that is a function of Ca^2+^ and a result which reflects a decrease in channel opening (34). While these opposing mechanisms of CDI loss reveal a difference in opening probability (P_O_) of Gly406Arg- and Gly402Ser-containing channels, both channel types do still suffer from a loss of VDI once they do open, and therefore both experience an increase in inward Ca^2+^ current, consistent with the model. It is important to mention that although the mechanism is straightforward in its effects on the cardiac AP, the mechanism in other tissues is not yet clear.

Expression of the variant Gly1911Arg in HEK293 cells affected VDI and steady-state inactivation (SSI), thereby significantly increasing the window current compared to WT cells (33). This mutant protein acts in the same way as the classic TS alleles in that it increases Ca^2+^ flux and thus causes a gain-of-function to the channel. The Gly1911 amino acid resides near the protein's C-terminus, distant from the positioning of other reported TS mutations. Its importance could be a result of its close proximity to a calcineurin binding site or its location on the distal C-terminus. Disruption of either of these domains could be related to impaired VDI (44, 45).

### Variants Resulting in an Alternative Mechanism: Gly419Arg, Arg518Cys/His, Ser643Phe, Glu1115Lys, Ile1166Thr

Ile1166Thr, which resides on the intracellular portion of S6 in the third repeat, was investigated in HEK293 cells (32). Functional studies revealed that unlike Gly406Arg and Gly402Ser, Ile1166Thr caused a large reduction in peak current density. Importantly, they also found that Ile1166Thr resulted in a negative shift in V_1/2_ of activation, or the degree of depolarization necessary for activation of the channel (32). Although less Ca^2+^ flows through the channel in a given opening event, the shift in activation and the resulting increase in window current confer Ile1166Thr with a net gain-of-function effect on CACNA1C channels. Wemhöner et al. also performed functional studies on this variant in HEK293 cells and *Xenopus* oocytes. Like Boczek et al., this group found that a negative shift in the V_1/2_ of activation was responsible for the gain of function conferred by Ile1166Thr. Additionally, Ile1166Thr does not affect VDI but does result in a decrease in steady-state inactivation (SSI) (11).

Boczek et al. performed functional studies on missense alleles Arg518Cys and Arg518His (27). When expressed in HEK293 cells, Arg518Cys resulted in a 55.6% decrease in peak current density. It also resulted in a decreased proportion of channels localized to the membrane vs. the cytoplasm, which could partially account for the reduction in overall Ca^2+^ flux. Arg518His caused a 63.2% decrease in peak current density and a negative shift in V_1/2_ of activation, similar to Ile1166Thr. Both variants caused a negative shift in V_1/2_ of inactivation as well, resulting in an increased window current and consequently, a net gain-of-function. Both Arg518Cys/His also resulted in decreased VDI, more similar to Gly406Arg/Gly402Ser than to Ile1166Thr (27).

Another variant with complex electrophysiological effects, Ser643Phe, was investigated in HEK293 cells (28). This variant resulted in an 86.2% reduction in peak current density. Once again, this variant caused a negative shift in activation and a large decrease in VDI, ultimately causing a net gain-of-function phenotype despite the dramatic reduction in Ca^2+^ flux (28).

The Gly419Arg variant acts uniquely when expressed in HEK293 cells (26). Despite actually having faster inactivation kinetics (a loss of function) than wild-type cells, peak current density is greatly increased. Like Ile1166Thr, Arg518His, Arg518Cys, and Ser643Phe, Gly419Arg resulted in a leftward, gain-of-function shift in the voltage dependence of activation leading to an increased window current for these channels (26).

Finally, the variant Glu1115Lys decreases Ca^2+^ flux in a unique way. When expressed in TSA201 cells, this variant converts the channel into a non-selective monovalent cation channel (30). There are four glutamate residues in the S4 helix that collectively form the ion selectivity filter. Altering one of these glutamate residues to a lysine alters the ion selectivity of the channel such that it becomes non-selective for monovalent cations. The result is a Cav1.2 channel with a slowly inactivating inward Na^+^ current and outward K^+^ current. The movement of these ions through a channel population not tuned to function in the cardiac action potential (AP) likely creates an environment in which arrhythmias are likely to develop (30).

## Discussion

### Future Research Directions

As one can see from the review of reported ATS variants, some are questionable as to whether they cause TS. Ideally, when new variants arise, it would be useful to carry out validation experiments such as reproducing the variant in human cardiomyocytes, showing that they are arrhythmogenic, and then correcting that variant back to the wild-type sequence. This would provide evidence that a given variant was responsible, at the very least, for the arrhythmic behavior of those cells. Assays don't yet exist to address how these variants specifically perturb the normal physiology of other tissues and organs in the body of a TS individual.

### *In vivo* Models

In addition to validating new variants, *in vivo* models will be useful for further investigation into the mechanism of TS. The various whole animal model studies were recently reviewed by Han et al. (46). Careful characterization of cell and animal models of all known TS variants is essential and will aid in any future development of treatments.

In brief, there are few animal models for TS. Knockout of the *CACNA1C* gene in mice is embryonic lethal (47) and heterozygotes are viable and fertile with no apparent developmental defects. There are no published reports of a TS1 mouse model other than a mouse line with restricted expression of a mutant *CACNA1C* gene under a craniofacial and limb bud mesenchymal promoter; such mice display craniofacial defects, specifically in the mandible (48). Defects in the limb buds were not described. This finding is interesting given that many TS1 children have mandible abnormalities and well as syndactyly.

As for TS2, animals expressing an inverted neomycin cassette were generated such that when the cassette was excised by recombination, a TS2 mutation would be expressed. When the cassette is properly recombined out, the homozygotes and heterozygotes are embryonic lethal; only heterozygous animals still bearing an inverted neomycin cassette inserted in the gene survive; these mice are referred to as TS2-neo mice. Presumably these mice survive because of reduced expression of the TS2 allele. The TS2-neo knock-in heterozygous mice have behavioral defects but no obvious cardiac defects (49, 50), however the authors do hypothesize that the TS2 mice lacking the neo cassette die due to cardiac defects. All these models were made before CRISPR gene editing technology was available. No other alleles have been made in the mouse to study any of the many ATS alleles described in this review.

Interestingly, no Timothy Syndrome *Drosophila* or zebrafish models have been described to date. Truncated alleles in zebrafish revealed a function for the Cav1.2 channel in embryonic heart development (51, 52) and pancreatic islets (53). A *C. elegans* TS animal was recently created by CRISPR; homozygous animals with the analogous G406R mutation (G369 in *C. elegans*) have widespread muscle defects and arrest as embryos or young larvae (54). No phenotypes were reported in heterozygous animals. One report of an ATS allele in *C. elegans* identified some axon targeting phenotypes (55) As there are an increasing number of ATS variants, an animal model that is amenable to inexpensive gene editing is needed to test their phenotypic consequences and investigate their mechanisms. Variants of Unknown Significance (VUS) can be verified in such models as well.

Besides the above models, there are just a few reports using human induced Pluripotent Stem Cells (hiPSCs). These are cells often collected from saliva or blood that can be induced to pluripotency, and then transfected with a cocktail of factors to induce them along a number of different cell fates. Cells collected from TS individuals have been used to study their arrhythmias in cardiomyocytes (CMs) derived from the hiPSCs (27, 56, 57). More recently, with the advent of CRISPR gene editing, “patient-independent” hiPSCs-CMs have been generated, speeding up the process in which cell lines can be generated. In such cases, numerous variants can be made from the same hiPSC control line such that the genetic background is isogenic (58, 59). Though hiPSCs have proved informative for TS and other LQT syndromes (59), it appears as if there is a rising interest in studying TS hiPSCs that have been driven to adapt neuronal fates (60–62). These approaches may highlight some of the neurodevelopmental defects that TS individuals experience.

### Nomenclature Proposal

Currently, gain of function variants in the *CACNA1C* gene are more likely to result in a diagnosis of non-syndromic LQT8. Variants in this gene have also been found to be associated with neuropsychiatric disorders such as schizophrenia (63, 64), bipolar disorder (65), and major depressive disorder (65). To date, mutations in a limited number of residues have been reported to cause TS.

The nomenclature for TS has been somewhat confusing in that TS1 and TS2 are caused by the same missense mutation but in two alternatively spliced exons. Though it has been argued that there is no clinical utility to distinguishing TS1 from TS2 (10), there is evidence that TS2 children have worse prognoses and differ from TS1 children in that they often develop hip dysplasia. Gly402Ser variants were originally also considered causative of TS2, which adds to the confusion. More recently, others have started to refer to this variant as causative of ATS. A recent proposal was made suggesting all mutations in the *CACNA1C* gene be considered TS1, and that TS2, TS3, and so on be reserved for other TS-causing genes yet to be discovered (17). After 17 years of TS molecular genetics, it seems unlikely that such other causative genes exist. We thus propose a more straight-forward nomenclature for the many TS variants that now exist. For historical reasons, TS1 and TS2 should remain as is, or perhaps TS1-Gly406Arg (exon 8A) and TS2-Gly406Arg (exon 8). We propose the remaining alleles be called ATS with the missense allele designation, such as ATS-Gly402Ser (exon 8) and ATS-Ser643Phe. Such names immediately indicate the molecular lesion in the *CACNA1C* gene. Though most ATS alleles are represented by a single case, these designations may prove useful in the future as additional children are identified as having one of these lesions. Alternatively, all the above case reports can be grouped as one syndrome, Timothy Syndrome, with the recognition that there is a broad and variable phenotypic spectrum.

### Diagnosis

In today's complex regulations involving health insurance, a diagnosis of TS means that a child is able to receive special services and benefits for their symptoms in multiple organs. Some of these services may include physical, occupational, behavioral, and speech therapy. Withholding such a diagnosis because an individual does not have the “classical” variant, or a “complete” TS phenotype, is not warranted at this time. Clinicians and researchers just do not understand this complex syndrome that well, and there are multiple sources of variability (as discussed earlier) that contribute to disease presentations.

### Natural History

The natural history of this syndrome is still being determined. One of us (KWT) has dedicated half her life to acquiring the medical records and family histories of over 60 children with TS, many of whom are no longer alive. To date, there are a number of young adults now in their late twenties thriving with TS. With more research into the progression of this ultra-rare syndrome, we hope that many more children can survive and live a long and productive life. Although much of the research to date has been on the cardiac consequences of these variants, there is an increasing interest in the neurodevelopmental phenotypes (42, 61, 62, 66). It is our hope that a greater number of researchers will also study the cellular mechanisms of these calcium channel variants that contribute to the multisystem nature of Timothy Syndrome (48, 67).

## Author Contributions

RB and AG wrote sections of the first draft of the manuscript. All authors contributed to overall outline of this review and manuscript revision. All authors read and approved the submitted version.

## Conflict of Interest

The authors declare that the research was conducted in the absence of any commercial or financial relationships that could be construed as a potential conflict of interest.
